# Patch-Based Texture Feature Extraction Towards Improved Clinical Task Performance

**DOI:** 10.3390/bioengineering12040404

**Published:** 2025-04-10

**Authors:** Tao Lian, Chunyan Deng, Qianjin Feng

**Affiliations:** 1 School of Biomedical Engineering, Southern Medical University, Guangzhou 510515, China; cuku078@163.com; 2 Guangdong Provincial Key Laboratory of Medical Image Processing, Southern Medical University, Guangzhou 510515, China; 3 Guangdong Province Engineering Laboratory for Medical Imaging and Diagnostic Technology, Southern Medical University, Guangzhou 510515, China

**Keywords:** patch-based texture features, radiomics, machine learning, medical image processing, cancer prediction

## Abstract

Texture features can capture microstructural patterns and tissue heterogeneity, playing a pivotal role in medical image analysis. Compared to deep learning-based features, texture features offer superior interpretability in clinical applications. However, as conventional texture features focus strictly on voxel-level statistical information, they fail to account for critical spatial heterogeneity between small tissue volumes, which may hold significant importance. To overcome this limitation, we propose novel 3D patch-based texture features and develop a radiomics analysis framework to validate the efficacy of our proposed features. Specifically, multi-scale 3D patches were created to construct patch patterns via k-means clustering. The multi-resolution images were discretized based on labels of the patterns, and then texture features were extracted to quantify the spatial heterogeneity between patches. Twenty-five cross-combination models of five feature selection methods and five classifiers were constructed. Our methodology was evaluated using two independent MRI datasets. Specifically, 145 breast cancer patients were included for axillary lymph node metastasis prediction, and 63 cervical cancer patients were enrolled for histological subtype prediction. Experimental results demonstrated that the proposed 3D patch-based texture features achieved an AUC of 0.76 in the breast cancer lymph node metastasis prediction task and an AUC of 0.94 in cervical cancer histological subtype prediction, outperforming conventional texture features (0.74 and 0.83, respectively). Our proposed features have successfully captured multi-scale patch-level texture representations, which could enhance the application of imaging biomarkers in the precise prediction of cancers and personalized therapeutic interventions.

## 1. Introduction

The volume, diversity, and rapid generation of medical imaging data for clinical diagnosis-decision are increasing exponentially. The non-invasive medical imaging offers comprehensive information to quantify intra-tumor heterogeneity and can reflect the genetic and cellular characteristics of tumors [1,2]. Radiomics, a prominent medical computer-aided technique, employs algorithms to decode medical image information and constructs predictive models for personalized diagnosis, and is gaining significant attention in research. Radiomics has predominantly been applied to cancer-related studies and has demonstrated significant success. Features appropriately extracted and quantified from medical images can support model development for tasks such as distinguishing cancer phenotypes or histological subtypes [3,4], predicting clinical staging [5], assessing treatment responses [6] and cancer genetics [7,8], predicting tumor metastasis risk [9], and forecasting survival [10]. The process of radiomics analysis includes volume of interest (VOI) segmentation, feature extraction, feature selection, and model construction.

Currently, the commonly utilized features in the field of radiomics include shape-based, intensity-based, texture-based, filter-based, and clinical features [11]. The intensity histogram (IH), describing the intensity distribution within an image, serves as a fundamental intensity-based feature. To integrate the intensity and spatial information, texture features such as Gray-Level Co-occurrence Matrix (GLCM) [12], Gray-Level Run Length Matrix (GLRLM) [13], Gray-Level Size Zone Matrix (GLSZM) [14], Neighborhood Gray Tone Difference Matrix (NGTDM) [15], filter-based features [16] (e.g., wavelet features, Laplacian of Gaussian (LOG) features), and Bag-of-Visual-Words (BoVW) [17] were introduced to complement IH. For instance, GLCM characterizes the co-occurrence probability of two voxels with specific intensities and spatial distribution across 26 directions in 3D space. In other words, GLCM is limited to describing patterns formed by two voxels. GLRLM counts the number of collinear runs where adjacent voxels share the same gray level across 26 directions in 3D space; thus, it serves as a descriptor of patterns aligned along lines. Similarly, GLSZM depicts the number of zones of a specific size sharing the same intensity within 26-connected neighborhoods across multiple sectional planes in 3D space. NGTDM captures the intensity difference between a central voxel and its surrounding voxels within a 26-connected neighborhood in 3D space. All these statistical feature matrices represent the distribution information among neighboring voxels. A voxel in a medical image typically represents a minuscule volume of tissue composed of cell clusters. Therefore, the aforementioned matrices are limited to describing voxel-based texture patterns. However, multi-scale patch patterns, which provide richer texture information related to extensive anatomic structures, have proven valuable for medical image segmentation [18], lesion detection [19], and lesion classification [20].

To capture large-scale contextual information, a commonly adopted approach is the use of a multi-resolution strategy [21]. Another approach involves using a set of voxels (such as voxel clusters in superpixels [22] or patches in BoVW-based methods) as the minimal unit for feature extraction, rather than individual voxels. Specifically, BoVW, which computes the occurrence probability of image patches within an image, has been extensively applied to medical image classification [23,24]. In BoVW, image patches are directly used to represent local visual patterns, with the scale of the pattern being determined by the patch size. Larger patches can represent larger-scale visual patterns; however, they often result in higher feature dimensionality. Therefore, balancing feature dimensionality and scale remains a critical challenge in medical image feature extraction [18]. From another perspective, BoVW can be considered an extended version of IH, where the patch in BoVW corresponds to the voxel in IH. While local visual patterns can be represented by patches, BoVW overlooks the spatial distribution information between patches. Inspired by BoVW and the progression from IH to statistical feature matrix-based methods such as GLCM, GLRLM, GLSZM, and NGTDM, this paper proposes a novel 3D patch-based texture feature extraction approach.

### Motivations and Contributions

Conventional texture features in medical image analysis predominantly focus on voxel-level statistical information, overlooking the spatial relationships between small tissue volumes that may contain significant clinical relevance. To address this limitation, this study proposes a novel approach that incorporates interaction information between small tissue patches within 3D space into conventional texture feature analysis. The main contributions of our work could be summarized as follows:

(1) We develop novel 3D patch-based texture features. Specifically, we construct patch patterns using k-means clustering and discretize images based on the labels of these patterns. The computation formulas of conventional texture features are modified to drive the extraction of patch-level (instead of voxel-level) texture features.
(2) In our proposed method, we design a multi-resolution framework via resampling image volumes to different voxel sizes and introduce multi-scale 3D patches to capture inter-patch correlation at different spatial scales simultaneously, thereby enabling a more comprehensive quantification of tissue characteristics.
(3) The machine learning models across five feature selection methods and five classifiers are performed to systematically validate the superiority and stability of the proposed 3D patch-based texture features relative to conventional texture features.
(4) Extensive experiments are conducted on simulated data and two independent MRI datasets involving three MRI sequences and two clinical prediction tasks to validate the effectiveness and generalizability of the proposed method over the conventional method.

## 2. Related Work

Compared with shape-based and intensity-based features, texture-related features play an important role in characterizing tissue heterogeneity. Aerts et al. [25] revealed that wavelet texture features, which capture different phenotypes of lung tumor computed tomography (CT) images, were significantly associated with the cell cycle pathway. GLCM and GLSZM have been shown to achieve superior survival prediction performance for high-grade osteosarcoma [26]. In PET imaging, a high baseline standardized uptake value (SUV) is often considered indicative of aggressive tumor behavior and poor prognosis [27,28]. However, SUV-based uptake values are insufficient for characterizing heterogeneous distribution [29]. Kido et al. [30] observed that the fractal features of bronchogenic carcinomas were significantly smaller compared to those of pneumonia and pulmonary nodules. In addition, many studies [31,32] have indicated that IH features derived from MR images are potentially useful for predicting cancer treatment outcomes. In [9,21,33], researchers demonstrated that textural features derived from matrices can more effectively predict the risk of tumor metastases. Petkovska et al. [34] showed that GLCM features can accurately distinguish malignant nodules from benign ones. Cook et al. [35] observed that NGTDM features were more effective than SUV measures in differentiating between responders and non-responders to chemoradiotherapy. In [36], LoG-filtered texture features extracted from CT images were shown to more effectively predict lung tumor stages beyond stage II. Based on these studies, it can be concluded that texture feature extraction is an essential step in the radiomics analysis. Therefore, improving the presentation ability of texture features is crucial for advancing radiomics applications and improving clinical outcomes.

## 3. Method

**Figure 1:**
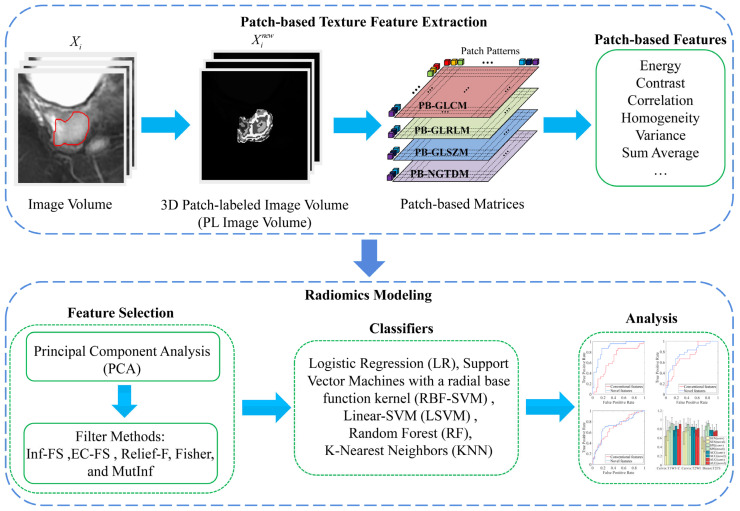
Overview of our workflow.

In this section, we provide a detailed description of our proposed approach. Figure 1 presents an overview of our workflow, which consists of two parts. The first part mainly consists of the generation of patch-labeled image volumes and the calculation of patch-based texture features, while the second part consists of a radiomics-driven cancer prediction designed to evaluate the effectiveness of the proposed method. The radiomics analysis includes tumor segmentation, image preprocessing, feature extraction, feature selection, and machine learning modeling.

### 3.1. Patch-Based Texture Feature Construction

#### 3.1.1. Patch-Labeled Image

In order to capture patch-level texture features in medical images, patch-labeled images (referred to as PL images) were created from the original images. This process involved three steps: (1) Three-dimensional patches were extracted from volume of interest (VOI) with each voxel as the center of a patch; (2) patch patterns (cluster centroids) were generated via k-means clustering from all extracted patches; (3) the voxel values of VOI were discretized by assigning the labels of patch patterns (cluster labels) based on the membership of each patch within predefined clusters. In this way, we can produce PL images. Next, we will elaborate on this process in detail.

Let X be a set of 3D images of m patients $X=\{X_{1},X_{2},...,X_{m}\}$, $X_{i}$ be a set of 3D patches of each patient $X_{i}=\{{\vec{x}}_{1},{\vec{x}}_{2},...,{\vec{x}}_{n}\}$ (n equals to the number of voxels in the VOI), and each patch be a vector ${\vec{x}}_{j}={\left\{p_{1},p_{2},...,p_{s}\right\}}^{T}$. Here, we used P to represent patch size. Specifically, as the radius of the patch increases, the length of the vector ${\vec{x}}_{j}$ increases. For the computation of patch patterns, an unsupervised clustering algorithm, specifically, k-means ($k_{m}$) with hard assignment, was applied for X to cluster patches into *k* clusters:

(1) $$\left[D,DL]=k_{m}(X\right)$$

The patterns dictionary $D=\{{\vec{d}}_{1},{\vec{d}}_{2},...,{\vec{d}}_{K}\}$ was produced, where ${\vec{d}}_{k}$ is the centroid of all patches belonging to the $k^{th}$ cluster and $DL=\{dl_{1},dl_{2},...,dl_{K}\}$ represents the labels of patterns. In the next step, a projection function of P was used to project each image $X_{i}$ to dictionary D:

(2) $$PBL_{i}=P(X_{i},D,DL)$$

where $PBL_{i}=\{pbl_{1},pbl_{2},...,pbl_{n}\}$ is the label set of patch patterns, with each $pbl_{j}$ representing a patch pattern label corresponding to the center of patch ${\vec{x}}_{j}$ in $X_{i}$. To calculate $pbl_{j}$, it is crucial to find the closest ${\vec{d}}_{k}$ in D for each ${\vec{x}}_{j}$; here, we explored the minimum value of function $min_{k=1,2,...,K}{\left\|{\vec{x}}_{j}-{\vec{d}}_{k}\right\|}_{2}$ to find the corresponding ${\vec{d}}_{k}$ and $dl_{k}$ of ${\vec{x}}_{j}$. Then, $pbl_{j}=dl_{k}$. Finally, PL image was generated by mapping $PBL_{i}$ to the corresponding position of patch ${\vec{x}}_{j}$ in $X_{i}$.

#### 3.1.2. Patch-Based Texture Features

Similar to the generation of conventional texture matrices, we utilized PL images and patch patterns to construct texture matrices for all patients. Further elaboration on the definition of texture matrices was provided in Appendix A. Meanwhile, we also revised the formulas of feature computation to more accurately represent the spatial distribution of patch patterns. A total of 39 novel patch-based texture features were extracted in this study (as listed in Table 1).

In patch-based GLCM features, $P\left(i,\;j\right)$ represents the number of instances of the $i^{th}$ patch pattern being neighbors with the $j^{th}$ patch pattern in VOI. Thus, gray-level i and j in the GLCM feature formulas are replaced with ${\vec{d}}_{i}$ or ${\vec{d}}_{j}$. Specifically, $\mu_{x}$ and $\mu_{y}$ are replaced by $\vec{\mu_{x}}$ and $\vec{\mu_{y}}$ to calculate the average values in every dimension of the patch pattern vector. $\sigma_{x}$ and $\sigma_{y}$ are substituted with covariance matrices of $\sigma_{x}$ and $\sigma_{y}$ to measure the fluctuant values of every dimension of patch pattern vector. In the contrast formula, ${\left(i-j\right)}^{2}$ is replaced with ${\left|{\vec{d}}_{i}-{\vec{d}}_{j}\right|}^{2}$ to describe the contrast and dissimilarity. In the homogeneity formula, we use ${\left|{\vec{d}}_{i}-{\vec{d}}_{j}\right|}^{2}$ to replace $\left|i-j\right|$ to simplified calculation. In the correlation and variance formula, $\left({\vec{d}}_{i}-\vec{\mu_{x}}\right)$ and $\left({\vec{d}}_{j}-\vec{\mu_{y}}\right)$ were used to replace $\left(i-\mu_{i}\right)$ and $\left(j-\mu_{j}\right)$, and the feature is extracted by solving the inner product or square. For the sum average, we use the mean $\vec{sum\;average}$ to depict the weighted sum of every dimension of the patch pattern vector.

Since GLRLM and GLSZM have similar feature formula structures, we use the same strategy to rewrite feature formulas. As with patch-based GLCM features, $\mu_{x}$ and $\mu_{y}$ are replaced with vectors $\vec{\mu_{x}}$ and $\vec{\mu_{y}}$, respectively, and gray-level i and j are replaced by ${\vec{d}}_{i}$ and ${\vec{d}}_{j}$, respectively. For formulas having $i^{2}$ in GLRLM and GLSZM features, we use ${\left|{\vec{d}}_{i}\right|}^{2}$ to replace $i^{2}$.

For patch-based NGTDM features, we use the same techniques used in other matrix features to revise formulas. In formulas of contrast, strength, and complexity, ${\left|{\vec{d}}_{i}-{\vec{d}}_{j}\right|}^{2}$ is used to replace ${\left(i-j\right)}^{2}$ or $\left|i-j\right|$ to reduce the complexity of computing. For feature busyness, vector $\vec{busyness}$ is computed to replace busyness, providing a more precise description of the feature’s characteristics. The revised formulas are demonstrated in Appendix A.

### 3.2. Radiomics Analysis Framework

#### 3.2.1. Segmentation of VOI

Accurate segmentation of tumor volumes is a critical step in the radiomics workflow. Although numerous semi-automatic and automatic segmentation methods exist, manual delineation remains a straightforward and reliable solution. To minimize inter-observer variability, two radiologists with over 10 years of interpretation experience were recommended to perform the VOI delineation. The segmentation results for each tumor were further validated, and the final segmentations were determined by a senior radiologist.

#### 3.2.2. Image Preprocessing

The intensity of images is normalized to reduce variations and facilitate subsequent calculations. A wavelet band-pass filter (WBPF) is applied for image denoising [21]. The ratios of weights in band-pass sub-bands to low- and high-frequency sub-bands were set to predefined values and denoted as R.

The inherent properties of matrix-based textural features render them highly sensitive to voxel size anisotropy. Therefore, a cubic interpolation algorithm [37] was employed to resample all images to isotropic voxel sizes with symmetric characteristics. Since images at different scales contain diverse information, a multi-resolution framework was incorporated in the proposed model. In a multi-resolution framework with S levels, images were resampled from the finest level $L_{0}$ to the coarsest level $L_{S-1}$:

(3) $${X^{\prime }}_{is}=\mathrm{Resampling}(X_{i},L_{s})$$

where $X_{i}$ is the $i^{th}$ 3D image, $L_{s}=\{L_{0},L_{1},...,L_{S-1}\}$ is the image scale level set, and ${X^{\prime }}_{is}$ is the resampled image at level $L_{s}$.

#### 3.2.3. Texture Feature Extraction

Based on multi-resolution images and multi-scale patches, PL images were computed from ${X^{\prime }}_{i}$ (${X^{\prime }}_{i}=\{{X^{\prime }}_{i0},{X^{\prime }}_{i1},...,{X^{\prime }}_{i(S-1)}\}$), obtained in (3) using the proposed algorithm. Then, the texture matrices were calculated from each specific-resolution PL image for each patient. Next, the novel second-order and high-order texture features, as shown in Table 1, were extracted from each matrix category. Consequently, a total of 39×R×S×P×K features were extracted for each patient.

#### 3.2.4. Feature Selection

Because the correlation and redundancy among features would inevitably affect classification performance, two steps of feature selection were performed in this study. In the first stage, we applied principal component analysis (PCA) [38] to reduce feature dimensionality, which can be regarded as an initial rough screening. The top 50 principal components with larger variances were kept in this stage. In the second stage, five well-known feature selection methods based on filter approaches were used: Inf-FS, EC-FS, Relief-F, Fisher, and MutInf [39]. Here, filter-based feature selection approaches were employed, as they are more efficient, less prone to overfitting than wrapper and embedded methods, and classifier-independent.

#### 3.2.5. Machine Learning Modeling

Single-source results from a certain classifier may have large deviations with the real results. We investigated five machine learning classifiers: Logistic Regression (LR), Support Vector Machines with a radial base function kernel (RBF-SVM), Linear-SVM (LSVM), Random Forest (RF), and K-Nearest Neighbors (KNN). The five feature selection and five classification methods were chosen due to their efficiency, simplicity, and popularity in the field of machine learning. The cross-combined performances of the five feature selection methods and five classification methods were compared. The accuracy (ACC), sensitivity (SEN), specificity (SPE), and area under the curve (AUC) from the receiver operating characteristic (ROC) curve were used to evaluate the classification performance.

#### 3.2.6. Clinical Utility

Radiomics serves as a bridge between medical imaging and personalized medicine. Developing effective biomarkers (radiomic features) constitutes one of the critical steps in radiomics analysis. In this study, we propose novel patch-based texture features with specific applications to MRI-based radiomics modeling for (i) axillary lymph node (ALN) metastasis prediction in breast cancer and (ii) histologic subtype prediction in cervical cancer.

Breast cancer and cervical cancer are common malignancies threatening women’s health. For breast cancer, ALN status is critical in determining the disease stage and predicting outcomes. Although pathological examination remains the gold standard for diagnosis, invasive biopsies may lead to complications such as pain and lymphedema. Studies have shown that 43–65% of patients with positive sentinel lymph nodes undergo unnecessary ALN dissection due to postoperative confirmation of non-metastatic ALN [40,41]. Similarly, adenocarcinoma (AC) and squamous cell carcinoma (SCC) are the primary histological subtypes of cervical cancer. Accurate histological subtyping and clinical staging are paramount for treatment planning and prognostication. For example, among locally advanced cervical cancer patients receiving radiotherapy or concurrent chemoradiotherapy, AC exhibits a worse prognosis compared to SCC [42].

Our approach proposes a non-invasive approach that may enable precise prediction of cervical cancer histological subtypes, assisting clinicians in optimizing treatment strategies to improve patient outcomes. Additionally, it provides a preoperative assessment tool for ALN metastasis status, potentially reducing unnecessary dissections and alleviating patient suffering. The experimental configurations are detailed in Section 4, and the results presented in Section 5 demonstrate the clinical utility of the proposed method over conventional texture features.

## 4. Materials and Experimental Configuration

### 4.1. Simulated Data

To illustrate the proposed novel patch-based texture features have stronger discriminative power than conventional features, two simulated images were generated. These simulated images consist of the same number of “+” and “−” symbols, while the arrangement of the symbols differs to represent distinct texture patterns between the two simulated images.

### 4.2. Clinical Data

This study was approved by our Institutional Review Board. The requirement for written consent was waived by the board. Two sets of MRI data were used in this study, one of which included 145 patients with breast cancer and information about axillary lymphatic metastasis. Imaging was conducted by using a 1.5T MR machine equipped with a four-channel breast coil in the prone position. The dataset consisted of T2-weighted fat suppression (T2FS) MR imaging modality (TR/TE = 3400/90 ms; FOV = 320 × 60 mm^2^; matrix = 348 × 299; slice thickness = 3 mm; slice gap = 0.3 mm). Among these patients, 55 had axillary lymphatic metastasis, while 90 had no metastasis. The results were confirmed through biopsy.

To demonstrate the effectiveness of the novel texture features in different clinical tasks, another MRI dataset comprising 63 patients with cervical cancer (squamous cell carcinoma or adenocarcinoma confirmed by pathology) was included in this study. MR imaging was performed before therapy using a 3.0T system, and pelvis examination was performed with a phased-array body coil. This dataset included a contrast-enhanced T1-weighted imaging sequence (T1WI+C) (TR/TE = 620/7 ms; FOV = 260 × 280 mm^2^; matrix = 256 × 256; slice thickness = 4 mm; slice gap = 6 mm; magnevist: 469 mg/mL; bodyweight: 0.2 mL/kg) and a T2-weighted imaging sequence (T2WI) (TR/TE = 3760/136 ms; FOV = 260 × 280 mm^2^; matrix = 256 × 256; slice thickness = 4 mm; slice gap = 6 mm). Among these patients, there were 39 patients with squamous cell carcinoma and 24 patients with adenocarcinoma.

### 4.3. Parameter Settings of Feature Extraction

The proposed method generates novel features based on four parameters: The ratio of weight in WBPF (*R*) which determines the proportion of band-pass sub-bands and the low/high frequency sub-bands; the multi-resolution image resampling level (*S*); the number of patch patterns in the dictionary (*K*); and the patch size (*P*). A summary of the parameter settings employed in this study is provided in the upper section of Table II. For parameter *S*, “pixelCS” refers to the physical pixel size in the cross-sectional image. For parameter *R*, ratios of 0.5, 1 and 1.5 were tested in this study. For parameters *S*, *K*, and *P*, various values were tested, and the ones yielding superior predictive performance were selected, as detailed in the upper part of Table 2.

### 4.4. Comparison of the Proposed Features with Conventional Features

To validate the superior performance of the novel texture features over conventional features, we conducted an experiment using the same model framework, differing only in the feature extraction part. Specifically, after applying the multi-resolution framework to the images, we employed two widely used quantization algorithms and a gray-level set (*N_g_*) to quantize the full intensity range of the tumor region. The parameter settings for the conventional features are presented in the lower half of Table 2. The quantization procedure maps voxel values to a finite set of discrete values. The two quantization algorithms are equal probability [43] and the Lloyd-Max quantization algorithm [44,45].

### 4.5. Division of Training and Validation Sets

All experiments were conducted using the five-fold cross-validation method. The dataset was divided into five subsets, ensuring an equal proportion of each class label in every subset. For each iteration, one subset was designated as the testing set, while the remaining four subsets were combined to form the training set. In this study, experiments were performed on each image modality from the two datasets, employing the developed radiomics analysis framework.

## 5. Results

### 5.1. Experiments on Simulated Data

**Figure 2:**
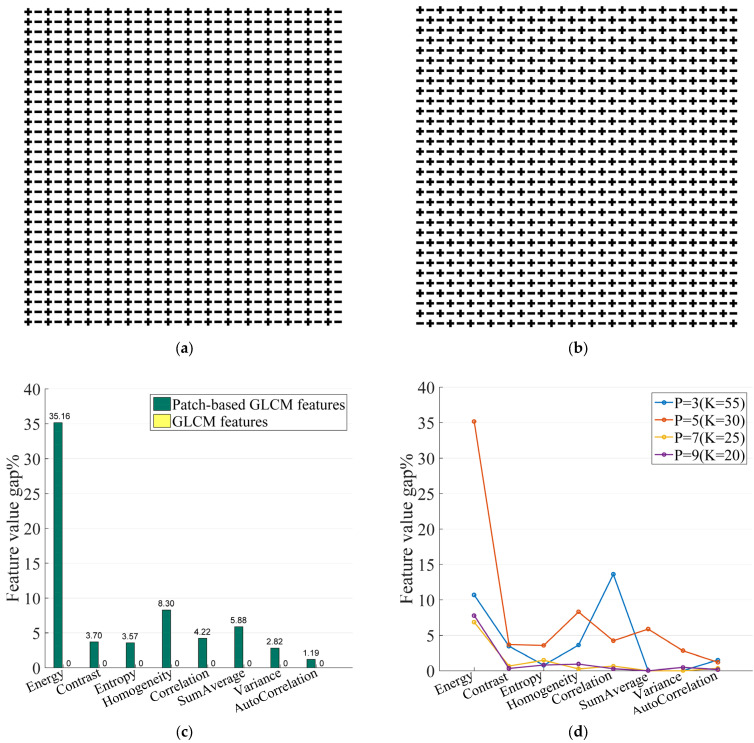
(**a**,**b**) are synthetic images. (**c**) GLCM-based feature value gaps between the synthetic images for the two methods. The y-coordinate values represent feature value gap ($y=\left|V_{\left(a\right)}-V_{\left(b\right)}\right|/V_{\left(a\right)}$ for each feature). (**d**) Feature value gap of the proposed patch-based GLCM features under different patch sizes. P = 5 and K = 30 are used to compute patch-based GLCM features.

Figure 2a,b shows the synthetic images. We computed the conventional GLCM features and proposed patch-based GLCM features from the two synthetic images, respectively. We observed that the two synthetic images are very similar. We computed the mean value of the GLCM-based features in four directions using a symmetric strategy. The feature differences between the two synthetic images are displayed in Figure 2c. It indicates that the conventional GLCM features could not demonstrate the difference between the two synthetic images, whereas the patch-based GLCM features could identify the difference. Specifically, the difference in the “energy” computed using the proposed method was significantly higher than that of the conventional method. Figure 2d depicts that the effect of adopting different patch sizes varies for each feature. We need to use different patch sizes to acquire more robust features.

### 5.2. Identification of the Optimal Combination

**Figure 3:**
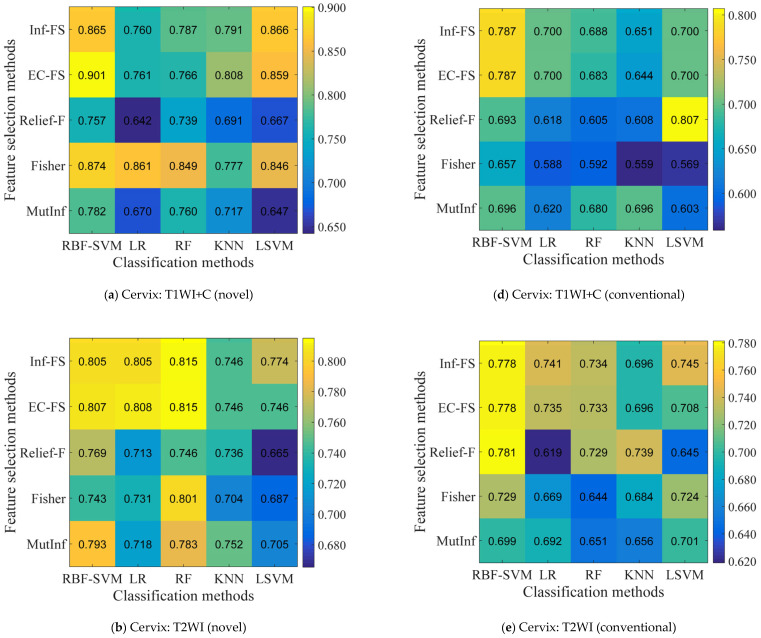

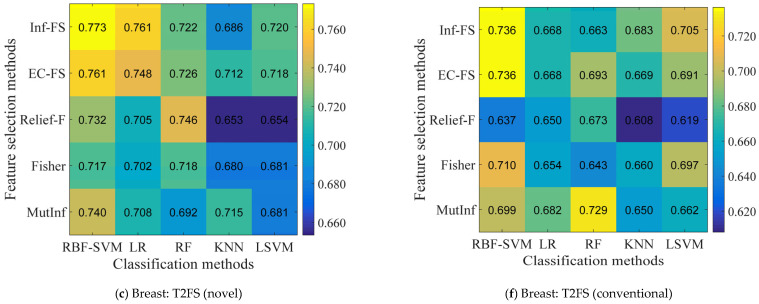
Heatmaps depict the prediction performance (AUC) of feature selection (in rows) and classification (in columns) methods. The best AUC values of 30 results for each combination method on different feature sources and modalities: the two rows represent novel features and conventional features results, respectively; the different columns represent different data modalities.

Twenty-five combinations of feature selection and classification methods were generated. For each combination, the number of selected features was set from 1 to 30 with an increase of 1. Subsequently, these subsets of selected features were fed into the classifiers. Figure 3 reports the highest AUC values via adjusting the selected feature numbers. It was observed that the combination “EC-FS + RBF-SVM” demonstrated superior prediction performance relative to other combinations across three imaging modalities for both novel and conventional features (average AUC: 0.823 vs. 0.767).

### 5.3. Influence of Feature Numbers on Prediction Performance

**Figure 4:**
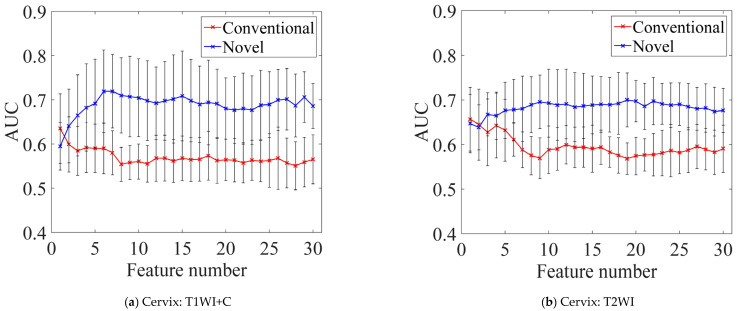

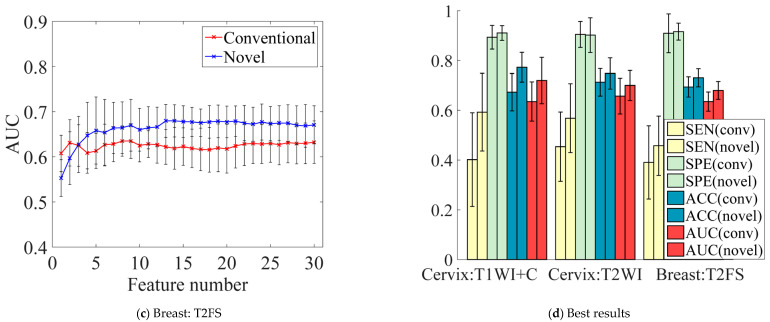
(**a**–**c**) The average AUC curves for 25 feature selection and classification methods for varying feature numbers in three modalities. (**d**) The best average performance across all combinations in three modalities.

To evaluate the impact of selected feature numbers on prediction performance, an integrated method was designed to compute the average AUC values across 25 combinations of feature selection and classification methods for each selected feature number (ranging from 1 to 30) for both conventional and novel features in all three modalities. As shown in Figure 4a–c, the average AUC values were stable with minor fluctuations when the feature number exceeded 10. Therefore, the first 30 features could represent a comprehensive feature set derived from the feature extraction process. Additionally, the best results among the 30 features in the integrated method were selected to compare the performance of conventional and novel features. Figure 4d indicates that the novel features outperform the conventional features across all three imaging modalities, as evidenced by the best SEN, SPE, ACC, and AUC values obtained in the integrated method.

### 5.4. Performance Comparison of Conventional vs. Novel Texture Features in the “EC-FS + RBF-SVM” Combination

**Figure 5:**
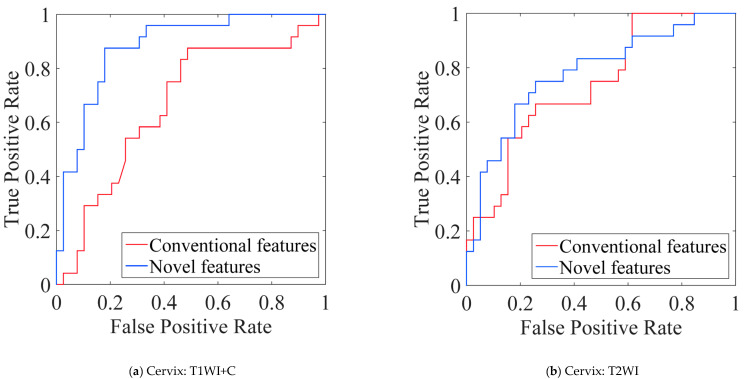

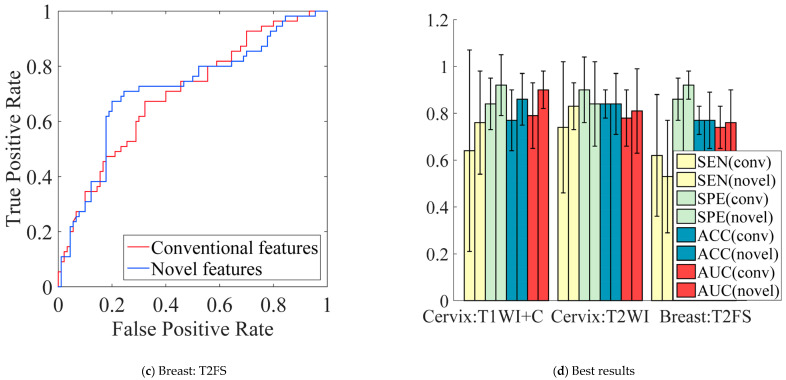
(**a**–**c**) The average ROC curves of conventional and novel features in three modalities using “EC-FS + RBF-SVM”. (**d**) The performance comparison between the proposed features and the conventional features by using “EC-FS + RBF-SVM” in three modalities.

To evaluate the effectiveness of the proposed novel features within a fixed combination of feature selection and classification, the “EC-FS + RBF-SVM” combination, known for its superior predictive performance, was selected. In each experiment, features were incrementally selected from 1 to 30 for classification using RBF-SVM, and the results with the maximum AUC value were recorded. Figure 5a–c indicates that the cross-validated average AUC values of the novel features outperform those of the conventional features across all three imaging modalities. The average SPE, SEN, and ACC values are shown in Figure 5d. Apart from SPE for subtype prediction of cervix cancer in T2WI and SEN for metastasis prediction of breast cancer in T2FS, the novel features also demonstrated more satisfactory results than the conventional features.

### 5.5. Performance Comparison of Conventional vs. Novel Texture Features in the Multi-Modality of Cervical Cancer

**Figure 6:**
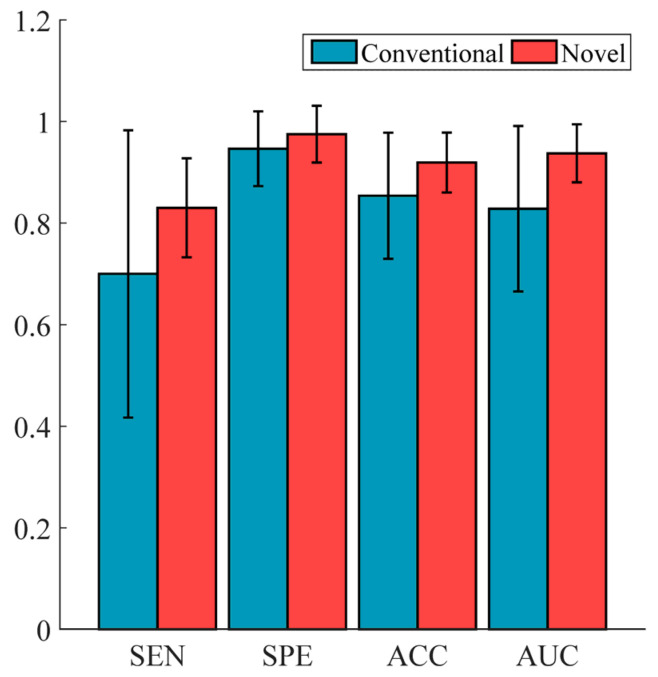
The performance comparison between the proposed features and the conventional features in the multiple modalities. The features extracted from T1WI+C and T2WI were combined and inputted into the “EC-FS + RBF-SVM” model.

Each imaging modality provides unique information; therefore, radiomics features derived from multiple modalities may encapsulate richer information and achieve superior predictive performance. In this study, we integrated the two imaging modalities of cervical cancer to evaluate the performance of the proposed novel features. Additionally, the “EC-FS + RBF-SVM” combination was employed to construct the model. Similarly, we selected the optimal results via adjusting feature count (ranging from 1 to 30) and calculated the average results of cross-validation as the final output. As shown in Figure 6, the proposed novel texture features demonstrated better predictive performance compared to conventional features. Compared to Figure 5d, the features derived from combined modalities outperformed those from individual modalities.

## 6. Discussion

Medical imaging in clinical oncology is routinely used and easily accessible [43]. Radiomics is an emerging and rapidly advancing field aimed at developing imaging biomarkers to enhance decision support in clinical practice [46,47]. Additionally, texture features play a critical role in radiomics analysis. The spatial distribution information reflecting the mutual interactions of small tissues can significantly influence radiomics-based analysis. However, conventional texture features fail to leverage this spatial information. Building on this observation, we proposed 3D patch-based statistical texture features. Synthetic experiments demonstrated that the proposed method effectively captured large-scale patterns and revealed their distribution relationships. However, determining the most effective tissue size remains a challenge. A fixed tissue size may not be suitable for different patients and tumors. To address this issue, we employed varying sizes of 3D patches and diverse numbers of 3D patch patterns for parallel feature extraction, enabling the acquisition of complementary and robust texture information. Furthermore, multi-resolution imaging was adopted to mitigate the computational complexity associated with using large 3D patches. Therefore, this approach enabled the acquisition of more efficient and clinically useful biomarkers.

Previous studies employed limited feature selection and classification methods [48,49], with some relying on 2D images for radiomics analysis. Such approaches may yield results with low reliability and fail to accurately identify true biomarkers. In this study, we proposed a 3D patch-driven robust texture feature model, employing a two-stage feature selection process that refines features from coarse to fine. Additionally, we explored 25 cross-combinations of feature selection and classification methods commonly applied in radiomics-based prediction and classification tasks. The experimental results presented in this study demonstrated the effectiveness of the proposed novel texture features over conventional statistical texture features. These findings suggest that the spatial distribution interactions between small tissues play a critical role in radiomics analysis.

It is worth noting that the high-throughput features (39 × *R* × *S* × *P* × *K*) were extracted to comprehensively quantify the heterogeneity of tumors in this study. To address the issue of model overfitting caused by the “curse of high dimensionality”, which arises from an over-abundance of features relative to sample size, the two-stage feature selection process was therefore developed. Firstly, PCA was used for initial dimensionality reduction, retaining the top 50 principal components. PCA is an efficient linear dimensionality reduction technique that transforms the original features into a lower-dimensional space while preserving the maximum variance. As an unsupervised method, it does not rely on class labels, making it less prone to overfitting during preliminary screening—a crucial advantage given our limited sample size (145 breast cancer cases and 63 cervical cancer cases). Importantly, the principal components with maximum variances can capture tumor biological heterogeneity and demonstrate stronger associations with clinical outcomes [50]. Secondly, the filter-based feature selection methods were employed to rank the features for refined selection by evaluating the correlation between features and class labels along with interdependencies among features (e.g., Inf-FS, EC-FS). Finally, the non-informative and redundant features were eliminated, and the experimental results demonstrated that the top 10 feature subset achieved complete predictive representation capability, with AUC values maintaining stable fluctuations (Variance < 0.0001) as the feature set expanded from 10 to 30 dimensions (see Figure 4a–c).

The cross-combinations of five feature selection methods and five classifiers, both widely used in the field of radiomics, were investigated to systematically validate the effectiveness of the proposed patch-based texture features relative to the conventional texture features. The filter-based methods were chosen due to their high computational efficiency, independence from classifiers, and resistance to overfitting. The classifiers used include linear methods (LR, LSVM) and non-linear methods (RBF-SVM, RF, KNN), indicating different types of decision boundaries. Moreover, Du et al. [51] compared the diagnostic performance of 42 cross-combinations derived from six filter-based selection methods and seven baseline classifiers for radiomics-based differentiation between recurrence and inflammation of nasopharyngeal carcinoma. Laajili et al. [52] applied 10 filter-based feature selection methods and six baseline classifiers to improve the classification performance of breast cancer nodules (benign or malignant status). Salmanpour et al. [53] reported that filter-based feature selection methods (ReliefA) combined with classification algorithms (RF) produced higher performance than other machine learning models for predicting levodopa medication status in Parkinson’s disease patients using radiomics and clinical features. Therefore, we believe that it is appropriate to use these 25 combinations to validate the effectiveness of the proposed methods, though there may be other approaches we have not considered. Future studies integrating the proposed novel features with advanced modeling algorithms to boost the prediction performance in different clinical tasks are our active pursuit.

In this study, applying the proposed texture features showed a trend toward improved prediction performance relative to conventional texture features (Figure 4d, Figure 5d and Figure 6). We used AUC, ACC, SEN, and SPE to measure the prediction ability. AUC provides a comprehensive assessment of the model’s discriminative ability across different thresholds; ACC indicates the overall prediction accuracy; SEN and SPE measure the model’s ability to identify positive and negative cases, respectively. Therefore, these metrics, which are commonly used as evaluation standards in medical classification tasks, are sufficient to claim the superiority of our proposed novel features over conventional texture features. Compared to conventional processing workflow, the key innovations underlying our approach can be primarily attributed to the following critical aspects. First, the conventional method discretizes images based on the grayscale values of voxels, while the proposed method uses labels corresponding to patch patterns for image discretization. Second, different from conventional methods, the statistical values in patch-based matrices represent inter-patch statistics rather than inter-voxel statistics. Third, the conventional texture feature formulas are modified to extract patch-level rather than voxel-level texture features.

Table 3 further lists the comparisons of this study with the published papers in the two clinical tasks. Liu et al. [54] used PET/CT radiomics-based machine learning methods to predict histological subtypes in cervical cancer, achieving an AUC of 0.851 and ACC of 0.915 for PET radiomics, and AUC of 0.513 and ACC of 0.661 for CT radiomics. Wang et al. [55] indicated that the radiomics model from five combined MRI sequences (AUC = 0.89; ACC = 0.81) exhibited better differentiation ability than any MRI sequence alone for differentiating subtypes of cervical cancer. In our study, conventional texture features produced similar performance (AUC = 0.828; ACC = 0.854) with the previous studies, while our proposed texture features depicted the highest AUC of 0.937 and ACC of 0.919. Moreover, our results for predicting axillary lymph node status (AUC = 0.76; ACC = 0.77) are comparable with a study by Chen et al. [56], which investigated the predictive ability of MRI-based deep learning features combined with clinicopathological predictors, showing an AUC of 0.71 and ACC of 0.75 in the same clinical task. Similarly, Wang et al. [57] reported an AUC of 0.810 and ACC of 0.765 for axillary lymph node metastasis prediction in breast cancer based on an MRI radiomics model, and the performances were further improved after integrating the multi-modality radiomics features with clinicopathological factors. Furthermore, Liu et al. [58] developed a deep learning framework for the automated extraction of ultrasound deep features. When integrated with conventional radiomic features and clinicopathological parameters, this framework achieved superior predictive performance (AUC = 0.914–0.952; ACC = 0.87–0.89). While deep learning techniques enable the automated discovery of task-relevant features via end-to-end training, the learned features lack clear physical or biological interpretability, rendering the models often perceived as unexplainable “black boxes.” Moreover, due to their massive parameters, training deep networks substantially increases the demand for sample size. It should be noted that our proposed mathematically defined texture features not only provide interpretability but also demonstrate superior predictive performance over conventional texture features in small-sample scenarios, as they capture multi-scale spatial heterogeneity across tissue patches. Future research will explore the relationship and complementary value between the proposed features and deep features to enhance clinical prediction performance.

Regarding the feasibility of the proposed 3D patch-based texture feature analysis in real-world clinical applications, translating this framework into real clinical settings requires consideration of multiple aspects: (1) Computational efficiency: the algorithm has been implemented in MATLAB R2022b and can process common medical image formats (such as DICOM). Computing all features for a typical 3D MRI tumor volume takes approximately 1-2 min (on a workstation with Intel Core i7 processor, 32 GB RAM), which is acceptable for non-real-time clinical decision support; (2) Workflow integration: the method can be integrated into existing radiology workflows as an automated post-processing step. Following radiologists’ tumor segmentation, the system extracts features and generates prediction results as supplements for radiology reports; (3) User interface: A user-friendly interface needs to be further developed to enable clinicians to visualize prediction results via reliability estimates and explore key predictive features through interactive displays; (4) Clinical validation pathway: Before widespread clinical application, the method needs to undergo the following validation steps: (a) multi-center retrospective studies to validate performance across imaging devices and protocols; (b) prospective observational studies to evaluate performance in real clinical environments; (c) randomized controlled trials to assess the impact on patient outcomes when the method is incorporated into clinical decision processes; (5) Cost-effectiveness analysis: the method may reduce medical costs by decreasing unnecessary invasive procedures (e.g., biopsies) and optimizing treatment choices. Future studies should include detailed cost-effectiveness analyses to support clinical adoption decisions; (6) Regulatory considerations: As a medical decision support tool, the method needs to comply with relevant regulatory requirements (e.g., U.S. Food and Drug Administration (FDA) or Conformité Européenne (CE) certification). Our development process has followed principles of reproducibility and transparency, which will help meet these regulatory requirements. In conclusion, while translating our method into routine clinical practice requires further validation and development work, its technical feasibility, workflow compatibility, and potential to improve patient care make it a promising clinical decision-support tool.

Although the proposed study yielded reasonable results, it has certain limitations. First, a key limitation of this study lies in the absence of biological validation for the patch-based texture features, primarily due to the unavailability of histopathological and genomic correlation data at this stage. Future studies could address this gap by integrating relevant datasets to enhance the reliability of these features. In addition, the sample size for our datasets was not very large, it is essential to acquire additional patients and medical imaging modalities in a broader range of cancers to comprehensively validate the efficacy of the proposed method. Owing to variations in imaging device models and inconsistencies in imaging protocols, future studies should conduct a thorough evaluation of the robustness of the proposed novel features in multi-center settings.

In conclusion, we proposed 3D patch-based texture features and developed a radiomics analysis framework for predicting the histological subtypes of cervical cancer patients and axillary lymph node metastasis of breast cancer patients. Experimental results demonstrated that the proposed texture features outperformed conventional texture features in terms of AUC, ACC, SEN, and SPE across three MRI modalities in the two different clinical tasks. Furthermore, this study offers valuable insights into clinical decision-making. However, certain limitations remain, such as the need to collect additional data to further validate the robustness and generalization ability of the proposed method.

## Figures and Tables

**Table 1:** Groups of 3D patch-based texture features used in this study.

Texture Type	Texture Feature Description
Patch-based GLCM	Energy Contrast Correlation Homogeneity Variance Sum Average Entropy Auto Correlation
Patch-based GLRLM	Short Run Emphasis (*SRE*) Long Run Emphasis (*LRE*) Gray-Level Non-uniformity (*GLN*) Run-Length Non-uniformity (*RLN*) Run Percentage (*RP*) Low Gray-Level Run Emphasis (*LGRE*) High Gray-Level Run Emphasis (*HGRE*) Short Run Low Gray-Level Emphasis (*SRLGE*) Short Run High Gray-Level Emphasis (*SRHGE*) Long Run Low Gray-Level Emphasis (*LRLGE*) Long Run High Gray-Level Emphasis (*LRHGE*) Gray-Level Variance (*GLV*) Run-Length Variance (*RLV*)
Patch-based GLSZM	Small Zone Emphasis (*SZE*) Large Zone Emphasis (*LZE*) Gray-Level Non-uniformity (*GLN*) Zone-Size Non-uniformity (*ZSN*) Zone Percentage (*ZP*) Low Gray-Level Zone Emphasis (*LGZE*) High Gray-Level Zone Emphasis (*HGZE*) Small Zone Low Gray-Level Emphasis (*SZLGE*) Small Zone High Gray-Level Emphasis (*SZHGE*) Large Zone Low Gray-Level Emphasis (*LZLGE*) Large Zone High Gray-Level Emphasis (*LZHGE*) Gray-Level Variance (*GLV*) Zone-Size Variance (*ZSV*)
Patch-based NGTDM	Coarseness Contrast Busyness Complexity Strength

**Table 2:** Summary of the parameter settings used in the proposed method.

Feature Source	Modality	Parameter			
		*R*	*S*	*K*	*P*
Novel features	Breast-T2FS	0.5, 1, 1.5	pixelCS, 1, 1.3, 1.5, 1.7, 2	32, 128, 160, 256	3, 5
	Cervical-T1WI+C	0.5, 1, 1.5	PixelCS, 1, 2, 3, 4, 5	64, 128, 192, 256	3, 5
	Cervical-T2WI	0.5, 1, 1.5	PixelCS, 1, 2, 3, 4, 5	64, 128, 192, 256	3, 5
		*R*	*S*	*Quan.algo*	*N_g_*
Conventional features	Breast-T2FS	0.5, 1, 1.5	PixelCS, 1, 2, 3, 4, 5	Equal, Lloyd	8, 16, 32, 64
	Cervical-T1WI+C	0.5, 1, 1.5	PixelCS, 1, 2, 3, 4, 5	Equal, Lloyd	8, 16, 32, 64
	Cervical-T2WI	0.5, 1, 1.5	PixelCS, 1, 2, 3, 4, 5	Equal, Lloyd	8, 16, 32, 64

**Table 3:** Comparisons of this study with the published papers in the two clinical tasks.

Clinical Task	Study	Modality	Method	Sample Size (Train/Validation)	Validation Performance
Breast cancer axillary lymph node metastasis prediction	This study	T2FS	Three-dimensional patch-based texture features	145 (5-fold CV)	AUC = 0.76; ACC = 0.77
	Chen et al. [56]	DWI-ADC + DCE-MRI	Deep learning features and clinicopathological factors	479 (366/122)	AUC = 0.71; ACC = 0.75
	Wang et al. [57]	MRI	Conventional radiomics features	379 (247/132)	AUC = 0.810; ACC = 0.765
		MRI + Mammography	Multi-modality radiomics features and clinical predictors	379 (247/132)	AUC = 0.892; ACC = 0.818
	Liu et al. [58]	Ultrasound	Deep learning features and conventional radiomics features	883 (621/262)	AUC = 0.914–0.952; ACC = 0.87–0.89
Cervical cancer histological subtype prediction	This study	T1WI+C + T2WI	Three-dimensional patch-based texture features	63 (5-fold CV)	AUC = 0.937; ACC = 0.919
	Liu et al. [54]	PET	Conventional radiomics features	168 (136/59)	AUC = 0.851; ACC = 0.915
		CT	Conventional radiomics features		AUC = 0.513; ACC = 0.661
	Wang et al. [55]	T2SAG + T2TRA + CESAG + CETRA + ADC	Conventional radiomics features	96	AUC = 0.89; ACC = 0.81

DCE-MRI: dynamic contrast-enhanced MRI; DWI-ADC: diffusion-weighted imaging-quantitatively measured apparent diffusion coefficient imaging; T2SAG: sagittal T2-weighted imaging; T2TRA: transverse T2-weighted imaging; CESAG: sagittal contrast-enhanced T1-weighted imaging; CETRA: transverse contrast-enhanced T1-weighted imaging.

## Data Availability

The data presented in this study are available on request from the corresponding authors due to privacy or ethical restrictions.

## Appendix A. Definition of Patch-Based Features

### Appendix A.1. Patch-Based Gray-Level Co-Occurrence Matrix Features (Patch-Based GLCM Features)

Let *P* define the GLCM. *P*(*i*, *j*) represents the number of times voxels with the value of *i* (label of patch pattern *i*) were neighbors with voxels with the value of *j* (label of patch pattern *j*) in *V*. *N_g_* represents the number of patch patterns. Only one GLCM of size *N_g_ × N_g_* is computed per volume *V* by simultaneously adding up the frequency of co-occurrences of all voxels with their 26-connected neighbors in 3D space (All voxels in *V* are considered as center voxels for calculation once). Owing to discretization length differences, neighbors at a distance of *h* voxels around a center voxel increase the GLCM by a value of *h* ($h=1,\sqrt{2},\sqrt{3}$). The entry (*i*, *j*) of the normalized GLCM is then defined as:

$$p(i,j)=\frac{P(i,j)}{\sum_{i=1}^{N_{g}}\sum_{j=1}^{N_{g}}P(i,j)}$$

Now, let us define:

$$\vec{\mu_{x}}=\sum_{i=1}^{N_{g}}\vec{d_{i}}\sum_{j=1}^{N_{g}}p(i,j),\;\;\vec{\mu_{y}}=\sum_{j=1}^{N_{g}}\vec{d_{j}}\sum_{i=1}^{N_{g}}p(i,j),\;\sigma_{x}=\sum_{i=1}^{N_{g}}[(\vec{d_{i}}-\vec{\mu_{x}}){\left(\vec{d_{i}}-\vec{\mu_{x}}\right)}^{T}]\sum_{j=1}^{N_{g}}p(i,j),\;\;\sigma_{y}=\sum_{j=1}^{N_{g}}[(\vec{d_{j}}-\vec{\mu_{y}}){\left(\vec{d_{j}}-\vec{\mu_{y}}\right)}^{T}]\sum_{i=1}^{N_{g}}p(i,j)\;p_{x}\left(i\right)=\sum_{j=1}^{N_{g}}p\left(i,j\right),\;\;p_{y}\left(j\right)=\sum_{i=1}^{N_{g}}p\left(i,j\right).$$

Then, the patch-based GLCM texture features are defined as:

(1) Energy:

$$energy=\sum_{i=1}^{N_{g}}\sum_{j=1}^{N_{g}}{\left[p\left(i,j\right)\right]}^{2}$$

(2) Contrast:

$$contrast=\sum_{i=1}^{N_{g}}\sum_{j=1}^{N_{g}}{\left|\vec{d_{i}}-\vec{d_{j}}\right|}^{2}p\left(i,j\right)$$

(3) Correlation:

$$correlation=\sum_{i=1}^{N_{g}}\sum_{j=1}^{N_{g}}\frac{{\left(\vec{d_{i}}-\vec{\mu_{x}}\right)}^{T}(\vec{d_{j}}-\vec{\mu_{y}})p(i,j)}{{\left[diag(\sigma_{x})\right]}^{T}diag(\sigma_{y})}$$

(4) Homogeneity:

$$homogeneity=\sum_{i=1}^{N_{g}}\sum_{j=1}^{N_{g}}\frac{p\left(i,j\right)}{1+{\left|\vec{d_{i}}-\vec{d_{j}}\right|}^{2}}$$

(5) Variance:

$$variance=\frac{1}{N_{g}\times N_{g}}\sum_{i=1}^{N_{g}}\sum_{j=1}^{N_{g}}\left({\left|\vec{d_{i}}-\vec{\mu_{x}}\right|}^{2}+{\left|\vec{d_{j}}-\vec{\mu_{y}}\right|}^{2})p(i,j\right)$$

(6) Sum Average:

$$sum\;average=mean\{\frac{1}{N_{g}\times N_{g}}\sum_{i=1}^{N_{g}}\sum_{j=1}^{N_{g}}\left[\vec{d_{i}}p(i,j)+\vec{d_{j}}p(i,j)\right]\}$$

(7) Entropy:

$$entropy=-\sum_{i=1}^{N_{g}}\sum_{j=1}^{N_{g}}p\left(i,j\right)\log_{2}\left[p\left(i,j\right)\right]$$

(8) Auto Correlation:

$$auto\;correlation=\sum_{i=1}^{N_{g}}\sum_{j=1}^{N_{g}}\left({\vec{d_{i}}}^{T}\vec{d_{j}}\right)p\left(i,j\right)$$

Here, $\vec{d_{i}}$ represents the column vector obtained by flattening patch pattern i, and $\vec{d_{j}}$ represents the column vector obtained by flattening patch pattern j. The calculation of conventional gray-level co-occurrence matrix features refers to the description in study [21].

### Appendix A.2. Patch-Based Gray-Level Run-Length Matrix Features (Patch-Based GLRLM Features)

Let *P* define the GLRLM. *P(i, j)* represents the number of runs of voxels with the value of *i* (label of patch pattern *i*) and of length *j* in *V*. *N_g_* represents the number of patch patterns. *N_r_* represents the length of the longest run of any label-level in *V*. Only one GLRLM of size *N_g_* × *N_r_* is computed per volume *V* by simultaneously adding up all possible longest run-lengths in the 13 directions of 3D space (One voxel could be part of multiple runs in different directions, but can be part of only one run in a given direction). Due to discretization length differences, runs constructed from voxels separated by a distance of *h* increment the GLRLM by a value of *h* ($h=1,\sqrt{2},\sqrt{3}$). The entry *(i, j)* of the normalized GLRLM is defined as:

$$p(i,j)=\frac{P(i,j)}{\sum_{i=1}^{N_{g}}\sum_{j=1}^{N_{r}}P(i,j)}$$

Let us define:

$$\vec{\mu_{i}}=\sum_{i=1}^{N_{g}}\vec{d_{i}}\sum_{j=1}^{N_{r}}p(i,j),\;\;\mu_{j}=\sum_{j=1}^{N_{r}}j\sum_{i=1}^{N_{g}}p(i,j)$$

Then, the patch-based GLRLM texture features are defined as:

(1) Short Run Emphasis (*SRE*):

$$SRE=\sum_{i=1}^{N_{g}}\sum_{j=1}^{N_{r}}\left[\frac{p\left(i,j\right)}{j^{2}}\right]$$

(2) Long Run Emphasis (*LRE*):

$$LRE=\sum_{i=1}^{N_{g}}\sum_{j=1}^{N_{r}}j^{2}p\left(i,j\right)$$

(3) Gray-level Non-Uniformity (*GLN*):

$$GLN=\sum_{i=1}^{N_{g}}{\left[\sum_{j=1}^{N_{r}}p\left(i,j\right)\right]}^{2}$$

(4) Run Length Non-Uniformity (*RLN*):

$$RLN=\sum_{j=1}^{N_{r}}{\left[\sum_{i=1}^{N_{g}}p\left(i,j\right)\right]}^{2}$$

(5) Run Percentage (*RP*):

$$RP=\frac{\sum_{i=1}^{N_{g}}\sum_{j=1}^{N_{r}}p\left(i,j\right)}{\sum_{j=1}^{N_{r}}j\sum_{i=1}^{N_{g}}p\left(i,j\right)}$$

(6) Low Gray-level Run Emphasis (*LGRE*):

$$LGRE=\sum_{i=1}^{N_{g}}\sum_{j=1}^{N_{r}}\left[\frac{p\left(i,j\right)}{{\left|\vec{d_{i}}\right|}^{2}}\right]$$

(7) High Gray-level Run Emphasis (*HGRE*):

$$HGRE=\sum_{i=1}^{N_{g}}\sum_{j=1}^{N_{r}}[{\vec{d_{i}}}^{T}\vec{d_{i}}]p\left(i,j\right)$$

(8) Short Run Low Gray-level Emphasis (*SRLGE*):

$$SRLGE=\sum_{i=1}^{N_{g}}\sum_{j=1}^{N_{r}}\left[\frac{p\left(i,j\right)}{{\left|\vec{d_{i}}\right|}^{2}j^{2}}\right]$$

(9) Short Run High Gray-level Emphasis (*SRHGE*):

$$SRHGE=\sum_{i=1}^{N_{g}}\sum_{j=1}^{N_{r}}\left[\frac{p\left(i,j\right){\left|\vec{d_{i}}\right|}^{2}}{j^{2}}\right]$$

(10) Long Run Low Gray-level Emphasis (*LRLGE*):

$$LRLGE=\sum_{i=1}^{N_{g}}\sum_{j=1}^{N_{r}}\left[\frac{p\left(i,j\right)j^{2}}{{\left|\vec{d_{i}}\right|}^{2}}\right]$$

(11) Long Run High Gray-level Emphasis (*LRHGE*):

$$LRHGE=\sum_{i=1}^{N_{g}}\sum_{j=1}^{N_{r}}p\left(i,j\right){\left|\vec{d_{i}}\right|}^{2}j^{2}$$

(12) Gray-level Variance (*GLV*):

$$GLV=\frac{1}{N_{g}\times N_{r}}\sum_{i=1}^{N_{g}}\sum_{j=1}^{N_{r}}{\left|\vec{d_{i}}p(i,j)-\vec{\mu_{i}}\right|}^{2}$$

(13) Run length Variance (*RLV*):

$$RLV=\frac{1}{N_{g}\times N_{r}}\sum_{i=1}^{N_{g}}\sum_{j=1}^{N_{r}}{\left(jp(i,j)-\mu_{j}\right)}^{2}$$

Here, $\vec{d_{i}}$ represents the column vector obtained by flattening patch pattern i. The calculation of conventional gray-level run-length matrix features refers to the description in study [21].

### Appendix A.3. Patch-Based Gray-Level Size Zone Matrix Features (Patch-Based GLSZM Features)

Let *P* define the GLSZM. *P*(*i*, *j*) represents the number of 3D zones of voxels with the value of *i* (label of patch pattern *i*) and of size *j* in *V*. *N_g_* represents the number of patch patterns. *N_z_* represents the size of the largest zone of any label-level in *V*. One GLSZM of size *N_g_ × N_z_* is computed per volume *V* by adding up all possible largest zone-sizes, with zones constructed from 26 connected neighbors of the same label-level in 3D space. One voxel can be part of only one zone. The entry (*i*, *j*) of the normalized GLSZM is defined as:

$$p(i,j)=\frac{P(i,j)}{\sum_{i=1}^{N_{g}}\sum_{j=1}^{N_{z}}P(i,j)}$$

Let us define:

$$\vec{\mu_{i}}=\sum_{i=1}^{N_{g}}\vec{d_{i}}\sum_{j=1}^{N_{z}}p(i,j),\;\;\mu_{j}=\sum_{j=1}^{N_{z}}j\sum_{i=1}^{N_{g}}p(i,j)$$

Then, the patch-based GLSZM texture features are defined as:

(1) Small Zone Emphasis (*SZE*):

$$SZE=\sum_{i=1}^{N_{g}}\sum_{j=1}^{N_{z}}\left[\frac{p\left(i,j\right)}{j^{2}}\right]$$

(2) Large Zone Emphasis (*LZE*):

$$LZE=\sum_{i=1}^{N_{g}}\sum_{j=1}^{N_{z}}j^{2}p\left(i,j\right)$$

(3) Gray-Level Non-uniformity (*GLN*):

$$GLN=\sum_{i=1}^{N_{g}}{\left[\sum_{j=1}^{N_{z}}p\left(i,j\right)\right]}^{2}$$

(4) Zone-Size Non-uniformity (*ZSN*):

$$ZSN=\sum_{j=1}^{N_{z}}{\left[\sum_{i=1}^{N_{g}}p\left(i,j\right)\right]}^{2}$$

(5) Zone Percentage (*ZP*):

$$ZP=\frac{\sum_{i=1}^{N_{g}}\sum_{j=1}^{N_{z}}p\left(i,j\right)}{\sum_{j=1}^{N_{z}}j\sum_{i=1}^{N_{g}}p\left(i,j\right)}$$

(6) Low Gray-Level Zone Emphasis (*LGZE*):

$$LGZE=\sum_{i=1}^{N_{g}}\sum_{j=1}^{N_{z}}\left[\frac{p\left(i,j\right)}{{\left|\vec{d_{i}}\right|}^{2}}\right]$$

(7) High Gray-Level Zone Emphasis (*HGZE*):

$$HGZE=\sum_{i=1}^{N_{g}}\sum_{j=1}^{N_{z}}{\left|\vec{d_{i}}\right|}^{2}p\left(i,j\right)$$

(8) Small Zone Low Gray-Level Emphasis (*SZLGE*):

$$SZLGE=\sum_{i=1}^{N_{g}}\sum_{j=1}^{N_{z}}\left[\frac{p\left(i,j\right)}{{\left|\vec{d_{i}}\right|}^{2}j^{2}}\right]$$

(9) Small Zone High Gray-Level Emphasis (*SZHGE*):

$$SZHGE=\sum_{i=1}^{N_{g}}\sum_{j=1}^{N_{z}}\left[\frac{p\left(i,j\right){\left|\vec{d_{i}}\right|}^{2}}{j^{2}}\right]$$

(10) Large Zone Low Gray-Level Emphasis (*LZLGE*):

$$LZLGE=\sum_{i=1}^{N_{g}}\sum_{j=1}^{N_{z}}\left[\frac{p\left(i,j\right)j^{2}}{{\left|\vec{d_{i}}\right|}^{2}}\right]$$

(11) Large Zone High Gray-Level Emphasis (*LZHGE*):

$$LZHGE=\sum_{i=1}^{N_{g}}\sum_{j=1}^{N_{z}}p\left(i,j\right){\left|\vec{d_{i}}\right|}^{2}j^{2}$$

(12) Gray-Level Variance (*GLV*):

$$GLV=\frac{1}{N_{g}\times N_{z}}\sum_{i=1}^{N_{g}}\sum_{j=1}^{N_{z}}{\left|\left(\vec{d_{i}}p(i,j)-\vec{\mu_{i}}\right)\right|}^{2}$$

(13) Zone-Size Variance (*ZSV*):

$$ZSV=\frac{1}{N_{g}\times N_{z}}\sum_{i=1}^{N_{g}}\sum_{j=1}^{N_{z}}{\left(jp(i,j)-\mu_{j}\right)}^{2}$$

Here, $\vec{d_{i}}$ represents the column vector obtained by flattening patch pattern i. The calculation of conventional gray-level size zone matrix features refers to the description in study [21].

### Appendix A.4. Patch-Based Neighborhood Gray Tone Difference Matrix Features (Patch-Based NGTDM Features)

Let *P* define the NGTDM. *P(i)* represents the sum of the magnitudes of the vector differences between the patch pattern vector of all voxels with the value of *i* (label of patch pattern *i*) and the mean patch pattern vector of its 26-connected neighboring voxels in 3D space. *N_g_* represents the number of patch patterns. One NGTDM of size *N_g_*×1 is computed per volume *V*. Like the matrices mentioned before, considering the discretization length differences, all averages around a center voxel located at position *(j, k, l)* in *V* are performed such that the neighbors at a distance of *h* voxels are given a weight of $1/\sqrt{h}\;$($h=1,\sqrt{2},\sqrt{3}$). The *P(i)* is defined as:

$$P(i)=\left\{\begin{array}{l} \sum_{voxels\in \left\{N_{i}\right\}}[({\vec{d}}_{i}-\vec{A_{i}}{)}^{T}({\vec{d}}_{i}-\vec{A_{i}})]\;\mathrm{if}\;N_{i}>0,\\ 0\;\mathrm{if}\;N_{i}=0. \end{array}\right.$$

where {*N_i_*} is the set of all voxels with label-level *i* in *V*, *N_i_* is the number of voxels with label-level *i* in *V*, and $\vec{A_{i}}$ is the average vector of patch patterns of the 26 connected neighbors around a center voxel with label value *i* and located at position *(j, k, l)* in *V,* namely:

$$\vec{A_{i}}=\vec{A}(j,k,l)=\frac{\sum_{m=-1}^{m=1}\sum_{n=-1}^{n=1}\sum_{o=-1}^{o=1}\omega_{m,n,o}\cdot \vec{d}(j+m,k+n,l+o)}{\sum_{m=-1}^{m=1}\sum_{n=-1}^{n=1}\sum_{o=-1}^{o=1}\omega_{m,n,o}},$$

where:

$$\omega_{m,n,o}=\left\{\begin{array}{l} 1\;\mathrm{if}\;\left|j-m\right|+\left|k-n\right|+\left|l-o\right|=1,\\ 1/\sqrt{2}\;\mathrm{if}\;\left|j-m\right|+\left|k-n\right|+\left|l-o\right|=2,\\ 1/\sqrt{3}\;\mathrm{if}\;\left|j-m\right|+\left|k-n\right|+\left|l-o\right|=3,\\ 0\;\mathrm{if}\;V(j+m,k+n,l+o)\;\mathrm{is}\;\mathrm{undefined}. \end{array}\right.$$

The quantity $n_{i}=\frac{N_{i}}{N}$ is also defined, where *N* is the total number of voxels in tumor volume.

Then, the patch-based NGTDM texture features are defined as:

(1) Coarseness:

$$coarseness={\left[\varepsilon +\sum_{i=1}^{N_{g}}n_{i}P(i)\right]}^{-1}$$

(2) Contrast:

$$contrast=[\frac{1}{N_{g}\times (N_{g}-1)}\sum_{i=1}^{N_{g}}\sum_{j=1}^{N_{g}}n_{i}n_{j}{\left|\vec{d_{i}}-\vec{d_{j}}\right|}^{2}]\times [\frac{1}{N}\sum_{i=1}^{N_{g}}P(i)]$$

(3) Busyness:

$$busyness=\frac{\sum_{i=1}^{N_{g}}n_{i}P(i)}{\sum_{i=1}^{N_{g}}\sum_{j=1}^{N_{g}}{\left|\vec{d_{i}}n_{i}-\vec{d_{j}}n_{j}\right|}^{2}},n_{i}\neq 0,n_{j}\neq 0$$

(4) Complexity:

$$complexity=\sum_{i=1}^{N_{g}}\sum_{j=1}^{N_{g}}\frac{{\left|\vec{d_{i}}-\vec{d_{j}}\right|}^{2}\left[n_{i}P(i)+n_{j}P(j)\right]}{N(n_{i}+n_{j})},n_{i}\neq 0,n_{j}\neq 0$$

(5) Strength:

$$strength=\frac{\sum_{i=1}^{N_{g}}\sum_{j=1}^{N_{g}}(n_{i}+n_{j}){\left|\vec{d_{i}}-\vec{d_{j}}\right|}^{2}}{\varepsilon +\sum_{i=1}^{N_{g}}P(i)},n_{i}\neq 0,n_{j}\neq 0$$

where ε is a small number to prevent feature values becoming infinite.

Here, $\vec{d_{i}}$ represents the column vector obtained by flattening patch pattern *i*, and $\vec{d_{j}}$ represents the column vector obtained by flattening patch pattern *j*. The calculation of conventional neighborhood gray-tone difference matrix features refers to the description in study [21].
